# Biohybrid M13 phage backpacks for active blood–brain barrier traversal and precision central nervous system leukemia treatment

**DOI:** 10.1186/s40364-026-01000-3

**Published:** 2026-09-16

**Authors:** Wei Wu, Cheng-Ling Zhang, Yu-Ling Yang, Xiaoyuan Chen, Xi Zhang, Xue Dong

**Affiliations:** 1 Medical Center of Hematology, Xinqiao Hospital of Army Medical University, Chongqing, 400037 P. R. China; 2 Chongqing Key Laboratory of Hematology and Microenvironment, Chongqing, 400037 P. R. China; 3 State Key Laboratory of Trauma and Chemical Poisoning, Chongqing, 400037 P. R. China; 4 School of Life Science, Chongqing University, Chongqing, 400044 P. R. China; 5 Shandong Provincial Key Laboratory of Precision Oncology, Shandong Cancer Hospital and Institute, Shandong First Medical University, Shandong Academy of Medical Sciences, Jinan, 250117 China

**Keywords:** Bacteriophage, Living materials, Biomimetic nanomaterials, Blood-brain barrier, Central nervous system leukemia

## Abstract

Central nervous system (CNS) leukemia remains a major clinical challenge in acute lymphoblastic leukemia, largely due to the restrictive nature of the blood–brain barrier (BBB), which severely limits therapeutic delivery and contributes to high relapse rates and poor prognosis. Here, we report ALB-phage, a bioengineered **a**nti-**l**eukemia **b**ackpack–phage system designed for active BBB navigation and intracranial drug delivery. M13 bacteriophages were genetically engineered to display BBB-homing ligands, forming a high-aspect-ratio biological transport platform (BTP). A leukemia-targeting therapeutic backpack (L-Pak) was constructed by encapsulating methotrexate within lipid nanoparticles fused with leukemia cell membranes, enabling homotypic targeting. The L-Pak was conjugated to the BTP via a matrix metalloproteinase-responsive PLGVR linker, allowing selective cleavage within the cerebrospinal fluid. Owing to its filamentous morphology, ALB-phage achieved efficient BBB translocation predominantly through energy-dependent macropinocytosis with an additional contribution from clathrin-mediated endocytosis. Both in vitro and in vivo studies demonstrate that ALB-phage effectively penetrates the BBB and selectively eradicates leukemia blasts, resulting in marked tumor suppression and a significant extension of median survival from 29 to 40 days in cell-line-derived xenograft models. Collectively, this M13 bacteriophage-based all-in-one platform integrates targeted BBB penetration with homology-mediated cytotoxicity, offering a promising strategy for the treatment of CNS leukemia and other central nervous system malignancies.

**Supplementary Information:**

The online version contains supplementary material available at https://doi.org/10.1186/s40364-026-01000-3.

## Introduction

Central nervous system (CNS) leukemia is a major cause of treatment failure and relapse in acute lymphoblastic leukemia (ALL) [1–4]. Nearly all ALL subtypes exhibit the capacity of leukemic cells to invade the subarachnoid space via vascular pathways from the bone marrow, turning the brain into a pharmacological sanctuary [5]. Approximately 30–70% of patients develop CNS involvement during disease progression, which markedly increases relapse risk and compromises long-term survival [6]. Chemotherapy, including high-dose methotrexate (HD-MTX) and intrathecal chemotherapy, remains the first-line treatment for CNS leukemia, aiming to increase drug accumulation in the cerebrospinal fluid (CSF) and brain parenchyma [7]. However, the inherent blood-brain barrier (BBB) limits the penetration of hydrophilic small molecules such as MTX, reducing CSF concentrations to 2–5% of plasma levels, and preventing effective anti-tumor activity [8, 9]. While intrathecal/intracerebral chemotherapy bypasses the BBB, the highly invasive treatment results in severe side effects, including infection, chemical arachnoiditis, and neurotoxicity, reducing the long-term compliance of patients. Therefore, developing non-invasive strategies for efficient and low-dose CNS drug delivery is essential to improve CNS leukemia treatment.

A variety of synthetic vehicles, including liposomes, polymers, and inorganic nanoparticles, have been engineered to enhance CNS delivery. Current strategies to overcome the BBB can be broadly categorized into three approaches: (1) Physical intervention, including focused ultrasound and X-ray irradiation, which temporarily and controllably disrupt BBB integrity [10–13]; (2) Biological hijacking, leveraging receptor-mediated transcytosis via peptides, proteins, cellular carriers, and cell membrane–mimetic nanovesicles for targeted drug delivery [14–18]; (3) Anatomical bypass, such as intranasal administration through the nose-to-brain axis [19, 20]. Although these strategies have shown promise for CNS tumors, their efficacy remains constrained by endogenous ligand competition, limited BBB transport efficiency, and rapid peripheral clearance, collectively impairing their therapeutic utility for CNS leukemia. Moreover, pronounced heterogeneity in CSF distribution restricts uniform drug exposure across diffusely infiltrating leukemic blasts, creating substantial pharmacological blind spots. Consequently, the development of novel delivery chassis capable of active barrier navigation and deep parenchymal penetration is critical to achieving effective intracranial anti-leukemic therapy while minimizing systemic toxicity.

Many pathogens have evolved intrinsic CNS tropism, enabling prolonged survival in the bloodstream and evasion of host immune surveillance. Recent advances demonstrate that biotic/abiotic hybrid nanosystems, using cells, bacteria, viruses, and their derivatives, can achieve synergistic treatments through tissue targeting, immune modulation, and metabolic regulation [21–23]. With its filamentous, high-aspect-ratio structure, the M13 bacteriophage offers several favorable features over synthetic nanocarriers, including programmable capsid proteins and an outstanding biosafety profile, making it a versatile platform for targeted delivery. Different from peptide-modified nanoparticles, the surface protein display system allows for the precise, high-density presentation of ligands, preserving their spatial configuration and accessibility. Furthermore, unlike traditional spherical nanoparticles, the long rod-like structure of the M13 bacteriophage provides substantial hydrodynamic advantages and endows it with a stronger ability to adhere to blood vessel walls and higher efficiency in penetrating the endothelium [24, 25]. We have previously shown that M13 bacteriophage crosses intestinal mucosal and lymphoid barriers [26, 27], suggesting its suitability as a biological chassis for blood–brain barrier penetration.

**Fig. 1:**
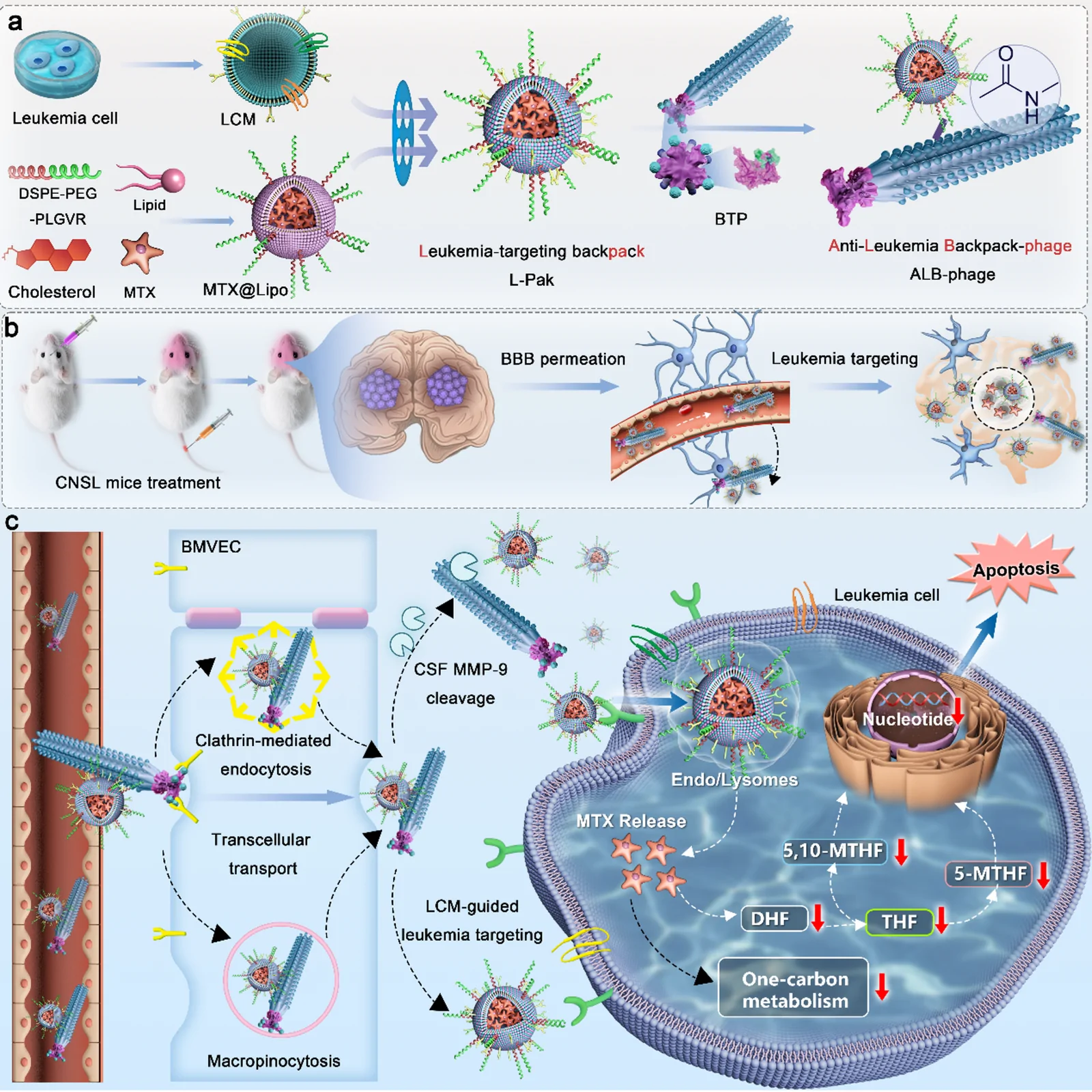
Schematic design and therapeutic mechanism of the ALB-phage for crossing the BBB and treatmenting CNS leukemia. (**a**) Preparation and assembly of the ALB-phage. MTX-loaded liposomes (MTX@Lipo) are camouflaged with LCM to form leukemia-targeting backpacks (L-Pak), which are subsequently conjugated onto brain-targeting phages (BTP) via specific linkers to obtain the ALB-phage system. (**b**) Schematic illustration of the systemic administration and in vivo delivery process of ALB-phage in CNS leukemia-bearing mice. (**c**) Cellular mechanism of BBB crossing and intracellular therapeutic process. ALB-phage crosses the BMVECs *via* multiple pathways, including clathrin-mediated endocytosis and macropinocytosis, to enable transcellular transport. After that, the leukemia-targeted backpack (L-Pak) is released in response to the CSF MMP-9 enzyme, and further homogeneously targets leukemia cells guided by LCM. The released MTX disrupts the one-carbon metabolism and folic acid metabolism to inhibit nucleotide synthesis and induce leukemia cell apoptosis

To enhance low-dose methotrexate (MTX) delivery to the CNS, we developed ALB-phage, a bioengineered Anti-Leukemia Backpack–phage system that employs a BBB-targeting M13 bacteriophage (BTP) as its navigational core (Fig. 1). By genetically engineering the pⅢ protein to display BBB-homing ligands, we endowed the BTP phage with active navigation ability, significantly enhancing its penetration efficiency across the BBB by controlling the spatial conformation and biological activity of the ligands. Moreover, the poor cell selectivity and slow metabolic clearance in the CNS of MTX raise concerns about neurotoxicity. To increase the selective killing effect of MTX on leukemia cells, a leukemia-targeting backpack (L-Pak) was developed by encapsulating MTX within lipid nanoparticles fused with leukemia cell membranes (LCM). Furthermore, using the peptide (PLGVR) responsive to cerebrospinal fluid matrix metalloproteinase (MMP) as a linker, L-Pak was conjugated to the BTP phage to obtain the bioengineered **A**nti-**L**eukemia **B**ackpack-phage (ALB-phage) (Fig. 1a-b). After intravenous injection, ALB-phage targets brain microvascular endothelial cells (BMVECs) and crosses the BBB *via* energy-dependent macropinocytosis. The local MMP-rich environment triggers the release of the L-Pak. The L-Pak was further guided by the specific surface molecules (CD7, ICAM1, and VCAM1) on the LCM, enabling the selective elimination of leukemia blasts through homotypic-mediated recognition (Fig. 1c). This strategy establishes an integrated delivery cascade, where the bacteriophage chassis facilitates active translocation, MMPs trigger site-specific release, and the LCM coating ensures precision ablation through homotypic recognition. Our results confirm that ALB-phage can effectively cross the BBB and specifically ablate ALL cells, significantly extending the median survival of cell-line-derived xenograft (CDX) models from 29 to 40 days (a 37.9% increase). This study establishes a novel, non-invasive strategy for precision therapy of central nervous system malignancies.

## Results and discussion

### Investigation of the brain-targeting and BBB-crossing effects of BTP bacteriophage

**Fig. 2:**
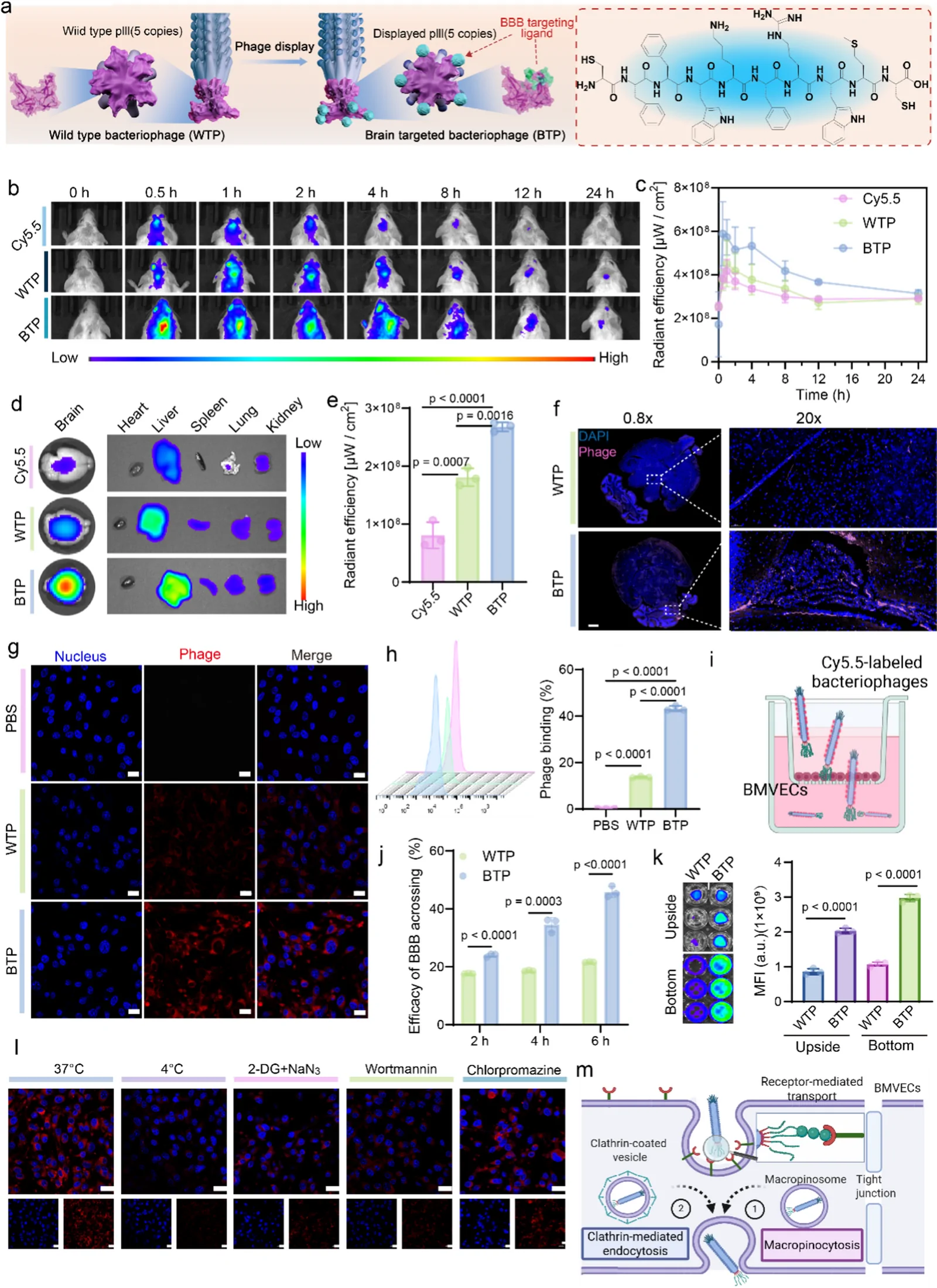
Evaluation of the enhanced brain-targeting ability by engineered BTP bacteriophage. (**a**) Schematic diagram of the M13 filamentous phages displaying a BBB-targeting peptide (BTP). (**b**-**c**) In vivo brain targeting capacity and biodistribution of BTP bacteriophage in CNS leukemia mice (*n* = 3). (**d**-**e**) Ex vivo fluorescence imaging and quantitative analysis of fluorescence intensity of brain tissues and major organs at 1 h (*n* = 3). (**f**) The representative fluorescent images of brain tissue after 1 h of injection of the Cy5.5-labeled bacteriophage (Scale bar: 1 mm in 0.8 × and 50 μm in 20 ×). (**g**) Typical CLSM fluorescence images of BMVEC cells after co-culturing with Cy5.5-labeled bacteriophages for 4 h (Scale bar: 50 μm). (**h**) Fluorescence intensity of Cy5.5-labeled BTP phage in BMVEC cells after 4 h incubation by FCM analysis (*n* = 3). (**i**) Schematic diagram of in vitro BBB model construction. (**j**) Fluorescence quantitative analysis of the efficiency of BTP crossing the BBB (*n* = 3). (**k**) The IVIS images of the fluorescence signals of BTP in the upper chamber and the lower chamber (*n* = 3). (**l**) Representative CLSM images of Cy5.5-labeled BTP after BMVEC treatment with different endocytosis inhibitors (*n* = 3) (Scale bar: 50 μm). (**m**) Mechanism diagram of BTP crossing the BBB. Data are presented as mean ± SD. Statistical analysis in (**e**, **h**, **k**) was determined by one-way ANOVA with Tukey’s post-hoc test, or in (**j**) was determined by multiple unpaired t-test

To enhance CNS-targeted penetration, the wild-type M13 bacteriophage (WTP) was genetically engineered via phage display to present the brain-targeting ligand CFFWKFRWMC [28] at the N-terminus of the pⅢ capsid protein (Fig. 2a and Supplementary Figure S1). The conjugation of the brain-targeting ligand to the N-terminus was confirmed by next-generation sequencing (NGS) (Supplementary Figure S2). Subsequently, the in vivo biodistribution of the engineered brain-targeting bacteriophage (BTP) was evaluated. Cy5.5-NHS was used to label both WTP and BTP. Meanwhile, free Cy5.5, a hydrophilic dye, served as a control. As shown in Fig. 2b-c, Cy5.5 exhibited a very short retention time in mouse brain tissue, beginning to clear approximately 1 h after intravenous injection. In contrast, mice treated with WTP displayed enhanced fluorescence signals and prolonged brain retention. The mice treated with BTP showed the strongest fluorescence signal in all the treatment groups, suggesting that the brain-targeting ligands significantly improved brain accumulation and retention. Ex vivo imaging of mouse organs and brain tissue confirmed these findings, showing that the brain-targeting efficiency of BTP was approximately 48% higher than that of WTP (Fig. 2d-e and Supplementary Figure S3). Brain tissue collected 1 h post-injection was subjected to immunofluorescence staining, which confirmed that BTP penetrated along the meninges into the brain parenchyma. The fluorescence intensity of BTP group in the brain was markedly higher than that of the WTP group (Fig. 2f). Next, we investigated the BBB-crossing pathway of the BTP bacteriophage. After co-culturing BMVEC with fluorescence-labeled bacteriophages for 4 h, confocal laser scanning microscope (CLSM) images showed that BTP displayed the strongest targeting effect on endothelial cells (Fig. 2g). Flow cytometry (FCM) analysis of endothelial cell uptake showed that BTP internalization was increased by 29.4% compared with WTP (Fig. 2h). Then, we established an in vitro BBB model using the Transwell chamber culture system to evaluate the transendothelial transport efficiency of BTP (Fig. 2i). Fluorescently labeled WTP and BTP were added to the upper chamber of a Transwell system, and solutions from the lower chamber were collected at specified time points for fluorescence measurement. As shown in Fig. 2j, the BBB-crossing efficiency of BTP increased progressively over time, whereas WTP exhibited limited translocation. Moreover, in vivo imaging system (IVIS) imaging revealed that the fluorescence intensity of BTP in both the upper and lower chambers was significantly higher than that of WTP, further demonstrating BTP’s enhanced ability to cross the BBB (Fig. 2k). Furthermore, the mechanism of transport responsible for the delivery of BTP across the BBB was demonstrated (Fig. 2l and Supplementary Figure S4). After low-temperature (4 °C) treatment, the endothelial cell transport ability of BTP significantly decreased, which was attributed to the attenuated intracellular signal transduction and endocytosis. Additionally, the endocytosis of BMVECs significantly decreased after treatment with 2-DG + NaN_3_ (energy-dependent inhibitor), indicating that the transendothelial transport of BTP was energy-dependent. In addition, a decrease in cell uptake was observed in the wortmannin (macropinocytosis inhibitor) and chlorpromazine (clathrin-dependent endocytosis inhibitor) treatment groups. Notably, the wortmannin treatment group showed the lowest level of cell uptake. Similarly, the fluorescence intensity of BTP in BMVECs was significantly decreased in the 2-DG + NaN_3_ and wortmannin treatment groups. Together, these results revealed that BTP crosses the BBB predominantly *via* energy-dependent macropinocytosis, with an additional contribution from clathrin-mediated endocytosis.

### Preparation and characterization of L-Pak

**Fig. 3:**
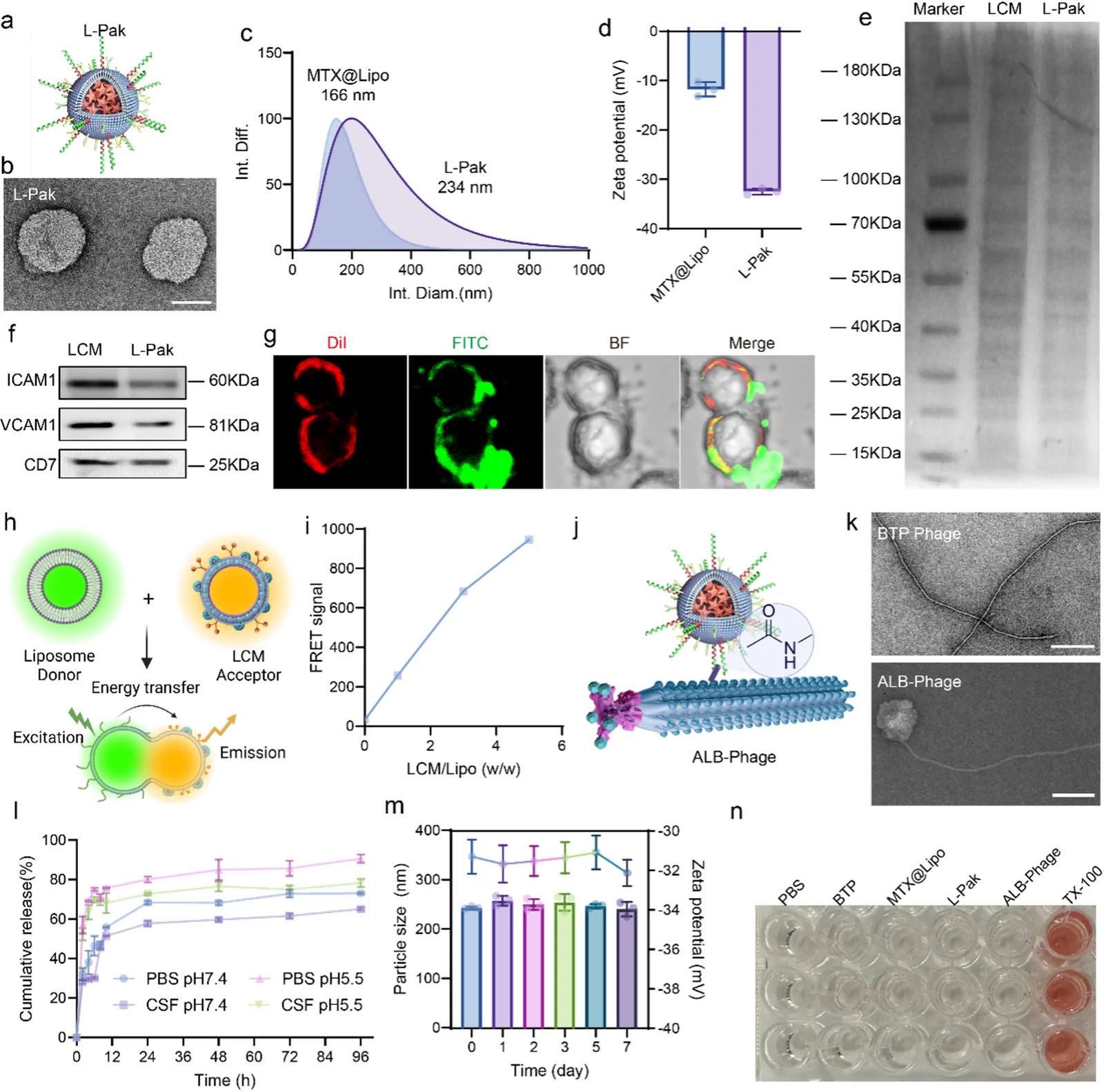
Preparation and characterization of ALB-phage. (**a**) Schematic diagram of the composition of nanovesicles. (**b**) TEM image of L-Pak (Scale bar: 100 nm). (**c**-**d**) Hydrolyzed particle size and zeta potential of MTX@Lipo and L-Pak (*n* = 3). (**e**) SDS-PAGE analysis of the protein components of LCM and L-Pak. (**f**) Western blot analysis of the characteristic membrane proteins (ICAM1, VCAM1, and CD7) in LCM and L-Pak. (**g**) Typical CLSM images of a fusion nanovesicle. Dil-labeled LCM, and MTX@Lipo were marked with FITC. (**h**-**i**) FRET assay and for the investigation of the fusion of LCM and Liposome. (**j**) Schematic diagram of BTP bacteriophage loaded with nanovesicle backpacks. (**k**) TEM images of BTP and ALB-phage (Scale bar: 200 nm in BTP phage and 100 nm in ALB-phage). (**l**) The release behavior of MTX in CSF and PBS at different pH values (*n* = 3). (**m**) Stability measurement of L-Pak and ALB-phage (*n* = 3). (**n**) Hemolysis test of different nanoparticles and the BTP-based system

Firstly, we prepared MTX-loaded liposomes (MTX@Lipo) via the hydration method. Then, the PLGVR peptide, which is responsive to the CSF matrix metalloproteinase-9 (MMP-9) [29, 30], served as a linker and was further incorporated into the nanovesicles (Fig. 3a and Supplementary Figure S5). To engineer a leukemia-targeting backpack, we further fused the leukemia cell membrane (LCM) with the liposomes (Lipo) through physical extrusion to obtain MTX-encapsulated nanovesicles (L-Pak) (Fig. 3a). Transmission electron microscopy (TEM) images revealed the spherical morphology of L-Pak (Fig. 3b). The dynamic light scattering (DLS) and Zeta potential results showed that the particle size of L-Pak was significantly larger than that of MTX@Lipo, and due to the fusion of membrane proteins, the surface electric potential was further reduced (Fig. 3c-d and Supplementary Figure S6). The increased size after hybridization revealed the integration of lipid bilayers from both LCM and Lipo. Furthermore, to confirm the fusion of the leukemia cell membranes, the protein components of the nanovesicles were confirmed through SDS-PAGE. As displayed in Fig. 3e, characteristic leukemia cell membrane proteins were detected in L-Pak. Additionally, western blot analysis revealed the presence of leukemia cell-specific markers [31], including CD7, ICAM1, and VCAM1 in the L-Pak (Fig. 3f). Moreover, fluorescence co-localization of Dil-labeled LCM and FITC-labeled Lipo displayed effective membrane hybridization (Fig. 3g). A Förster resonance energy transfer (FRET) study was also performed to evaluate the membrane fusion of LCM and Lipo. LCM and Lipo were hybridized at different ratios. FITC acted as the fluorescent donor while Dil served as the fluorescent acceptor. When the two membranes are close to each other, fluorescence energy will transfer from the donor to the acceptor. As shown in Fig. 3h-i, by increasing the ratio of LCM to Lipo, the FRET signals were enhanced, suggesting that the membrane fusion process between LCM and Lipo led to the close proximity of the fluorophore pair. These results collectively demonstrate the successful preparation of nanovesicles with selective targeting capabilities for leukemia cells. Subsequently, L-Pak was further loaded onto BTP through EDC/NHS conjugation (Fig. 3j). The TEM images and UV-vis showed that the M13 bacteriophage had a filamentous morphology, and L-Pak was successfully loaded onto the surface of BTP (Fig. 3k and Supplementary Figure S7). In addition, the release profiles of MTX in PBS and CSF were investigated in vitro at pH 7.4 and pH 5.5, respectively. As shown in Fig. 3l, under the physiological condition of pH 7.4, MTX in PBS exhibited a sustained and slow release. However, the release rate in CSF was relatively slow compared to the PBS group, which was attributed to the potential influence of the ionic concentration and buffering capacity of CSF on L-Pak. At pH 5.5, the release behavior of MTX was significantly accelerated due to the reduced stability of the nanovesicle membrane. Furthermore, the stability of ALB-phage was monitored in culture medium containing 10% FBS. As shown in Fig. 3m and Supplementary Figure S8, L-Pak exhibited slight variations in particle size and PDI, demonstrating its excellent stability. Meanwhile, a hemolysis assay was conducted to evaluate the biosafety of ALB-phage. There was no significant difference in the hemolysis rates of BTP, L-Pak, and ALB-phage, suggesting good hemocompatibility and safety (Fig. 3n).

### In vitro BTP-mediated L-Pak delivery and specific cytotoxicity

**Fig. 4:**
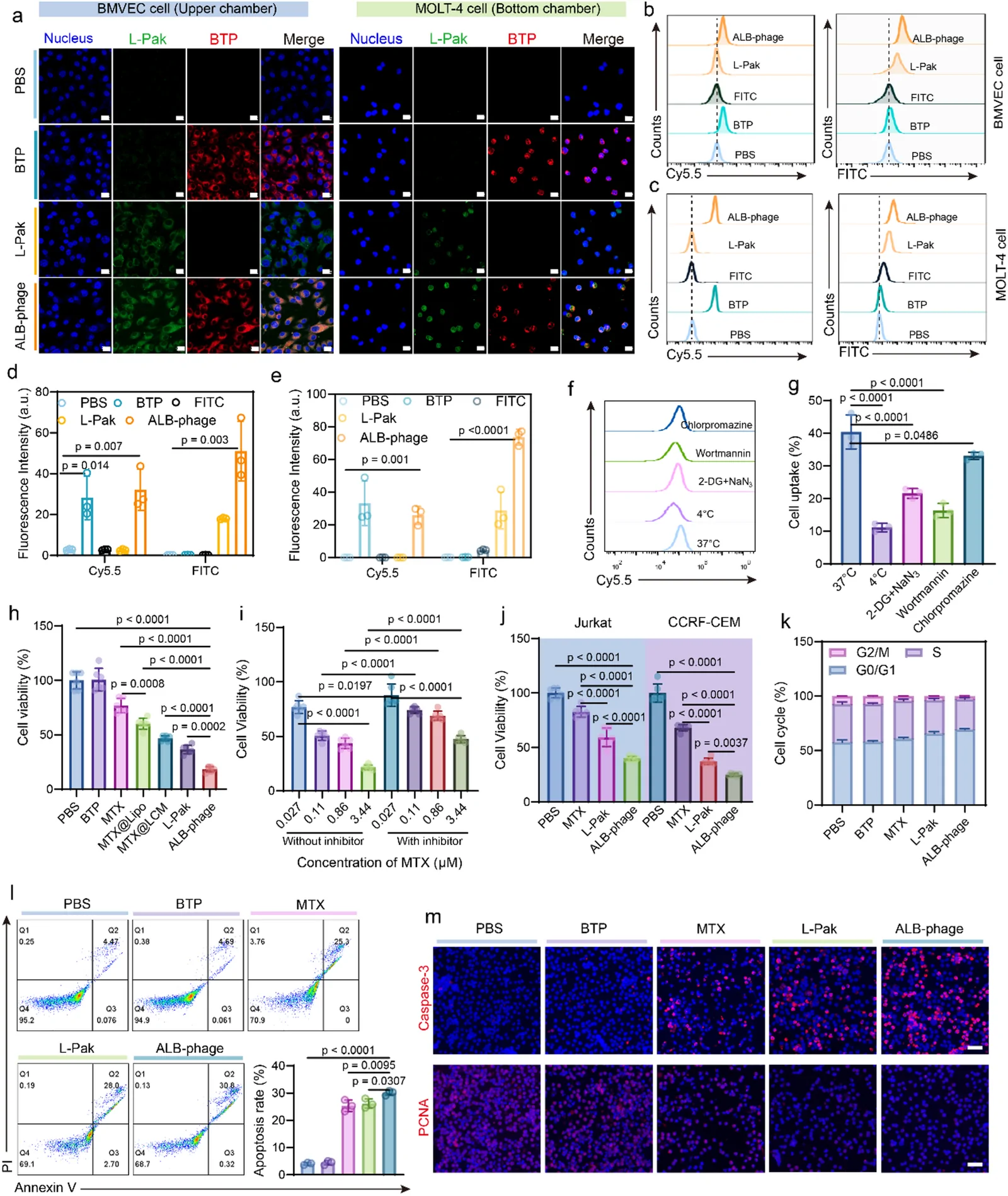
In vitro anti-leukemia effect of BTP-mediated transcellular transport of therapeutic doses of L-Pak. (**a**) Representative CLSM fluorescence images of BMVEC cells and ALL cells after co-culturing with Cy5.5-labeled bacteriophages for 6 h (Scale bar: 20 μm). (**b**-**e**) FCM and quantitative analysis of the levels of nanovesicle internalization by BMVEC and ALL cells after 6 h of incubation (*n* = 3). (**f**-**g**) The proportion of cellular internalization of Cy5.5-labeled L-Pak by BMVEC cells after various endocytosis inhibitor treatments (*n* = 3). (**h**) Cell viability of leukemia cells after different treatments (*n* = 6). (**i**) The cell viability of leukemia cells after treatment with ALB-phage combined with or without the MMP-9 enzyme inhibitor (*n* = 6). (**j**) Cell viability of Jurkat and CCRF-CEM cell lines after various treatments (*n* = 6). (**k**) Cell cycle arrest analysis after different treatments (*n* = 3). (**l**) The apoptotic level of leukemia cells after various treatments (*n* = 3). (**m**) Representative immunofluorescence images of leukemia cell apoptosis (Caspase-3) and proliferation (PCNA) (Scale bar: 50 μm). Data are presented as mean ± SD. Statistical analysis in (**g**, **h**, **i**, **j**, **l**) was determined by one-way ANOVA with Tukey’s post-hoc test, or in (**d**, **e**) was determined by multiple unpaired t-test

To demonstrate that BTP could also transport therapeutic nanovesicles across the BBB, we established an in vitro co-culture model of BMVECs and ALL cells. As shown in Fig. 4a, enhanced green fluorescence signals in both BMVEC cells and ALL cells were observed after treatment with ALB-phage. The L-Pak treatment group also demonstrated a certain ability for transendothelial transport, which was attributed to the BBB penetration mediated by the leukemia cell membrane. The FCM analysis of cell uptake also confirmed similar results (Fig. 4b-e). Both BTP and ALB-phage could cross the brain endothelial cell barrier and deliver the therapeutic L-Pak to ALL cells. Compared to BTP, the transendothelial barrier ability of ALB-phage was slightly reduced, mainly due to the increased size of the ALB-phage system. Meanwhile, an endocytosis inhibitor panel was used to determine the internalization pathways of ALB-phage. As expected, 2-DG + NaN_3_, chlorpromazine and wortmannin treatment groups showed decreased cellular uptake compared with the control group, suggesting both clathrin-dependent endocytosis and macropinocytosis were involved in the transcellular transport process of ALB-phage (Fig. 4f-g).

Subsequently, the cytotoxicity effect of ALB-phage on leukemia was evaluated. As displayed in Fig. 4h and Supplementary Figure S9-S10, BTP treatment showed no significant toxicity to leukemia cells. Due to the hydrophilicity of MTX, its cytotoxicity was limited at low concentrations. When further encapsulated in liposomes or cell membrane carriers, the cytotoxicity significantly increased, attributed to the enhanced cellular uptake and drug accumulation. Most notably, ALB-phage exhibited the strongest cytotoxicity against leukemia cells. The same result was confirmed by Calcein AM/PI staining (Supplementary Figure S11). Moreover, to demonstrate the CSF responsiveness of ALB-phage, we added the MMP inhibitor to block the release of L-Pak (Fig. 4i). As expected, Batimastat inhibitor treatment dramatically reduced cytotoxicity. ALB-phage, as a larger drug system, was difficult to effectively uptake by leukemia cells. Furthermore, BTP-mediated cytotoxicity in two different ALL cell lines was examined. As shown in Fig. 4j, ALB-phage demonstrated excellent cell-killing effects in both Jurkat and CCRF-CEM cell lines compared with L-Pak and MTX treatment groups. Similarly, due to the accumulation of enhanced MTX in leukemia cells, the cell cycle was arrested at the G1/S phase, resulting in a significant reduction in the proportion of the G2/M phase after ALB-phage treatment (Fig. 4k and Supplementary Figure S12). Consequently, the G1/S checkpoint blockade further induced the leukemia cells to undergo apoptosis (Fig. 4l), and the expression of anti-apoptotic proteins Bcl-2, the metabolic enzyme dihydrofolate reductase (DHFR), and cell cycle proteins (Cyclin D1 and CDK2) decreased correspondingly (Supplementary Figure S13). Meanwhile, immunofluorescence staining also revealed an enhanced signal of Caspase-3 in the ALB-phage-treated group, along with the lowest PCNA signal, suggesting effective cell apoptosis and proliferation inhibition (Fig. 4m). Collectively, these results elucidate the transendothelial transport pathway and specific targeting effects of BTP-guided nanovesicles, and also demonstrate the enhanced anti-leukemia effects of ALB-phage.

### In vivo brain targeting and biosafety of ALB-phage

**Fig. 5:**
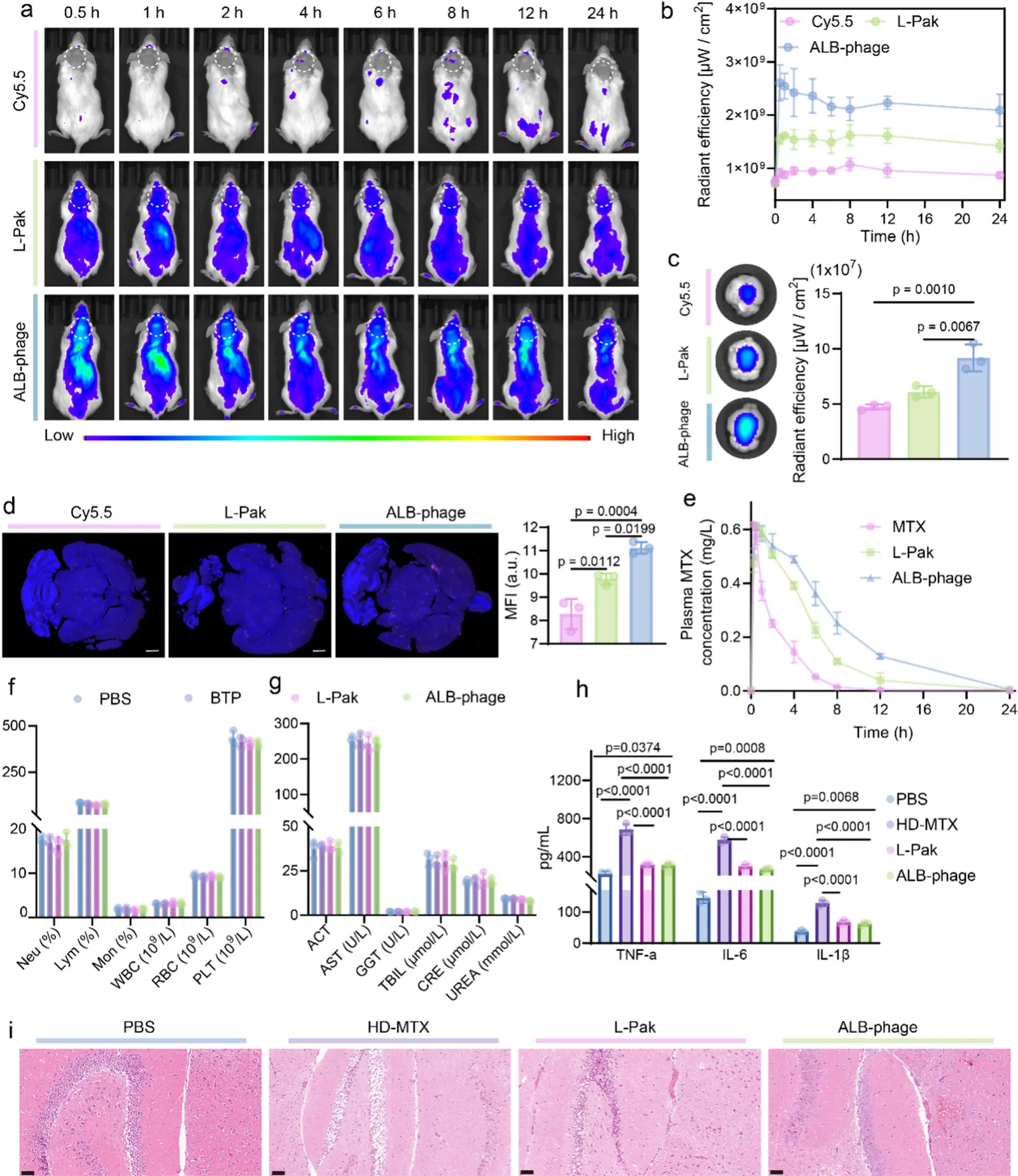
In vivo biodistribution and biosafety of ALB-phage. (**a**-**b**) The brain-targeting ability of ALB-phage in CNS leukemia mice and fluorescence quantitative analysis (*n* = 3). (**c**) Ex vivo fluorescence images of brain tissues and quantitative analysis after different treatments (*n* = 3). (**d**) Representative fluorescence images of brain tissues showing the penetration of Cy5.5-labeled L-Pak (Scale bar: 200 μm). (**e**) Analysis of the pharmacokinetics of MTX in PB after MTX, L-Pak and ALB-phage treatments (*n* = 3). (**f**-**g**) The blood routine and blood biochemical measurements for blood cells and liver and kidney function evaluation (*n* = 3). (**h**) Serum cytokine levels in healthy BALB/c mice after HD-MTX, L-Pak and ALB-phage treatments (*n* = 3). (**i**) H&E staining of the hippocampal region of the brain tissues after different treatments (Scale bar: 100 μm). Data are presented as mean ± SD. Statistical analysis in (**c**, **d**, **h**) was determined by one-way ANOVA with Tukey’s post-hoc test

The aforementioned results have preliminarily demonstrated the potential of BTP to transport nanovesicles across transendothelial barriers. Therefore, we further established an orthotopic CNS leukemia mouse model to evaluate brain targeting and in vivo biodistribution. Cy5.5-labeled L-Pak and ALB-phage were intravenously injected, and the biodistribution was monitored by the IVIS imaging system at the indicated time. As shown in Fig. 5a-b, no fluorescence signals were observed in the brain tissue after Cy5.5 treatment, indicating that hydrophilic small-molecule drugs have difficulty crossing the BBB and accumulating in the CNS. The L-Pak mediated by leukemia cell membranes showed a limited brain targeting effect. Meanwhile, ALB-phage exhibited significantly enhanced fluorescence signals in the brain and significantly prolonged the brain retention time compared with the L-Pak treatment group. After 6 h post-injection, the ex vivo fluorescence imaging of the mouse brain tissue also displayed intense fluorescence signals in the ALB-phage treatment group, with an increase of 50.4% compared to the L-Pak group (Fig. 5c and Supplementary Figure S14). To verify whether BTP could infiltrate the brain parenchyma, the mouse brain tissues were collected and evaluated through immunofluorescence staining. Consistent with the above results, a Cy5.5 fluorescence signal was observed both in the meninges and brain parenchyma after ALB-phage treatment, which was significantly higher than that in the L-Pak treatment group (Fig. 5d). Furthermore, the pharmacokinetics of MTX were investigated (Fig. 5e). The free MTX rapidly decreased in plasma concentration after 2 h post-injection. Conversely, increased blood circulation time was found in L-Pak and ALB-phage treatment groups due to encapsulation by carriers, thereby enhancing the bioavailability of MTX. Although bacteriophages are a type of pathogenic microorganism, our previous research indicates that M13 bacteriophages are a safe living carrier. To strengthen the clinical potential of the M13 bacteriophage, acute liver and kidney toxicity were further evaluated (Fig. 5f-g). There were no significant changes in blood cells and liver/kidney function among the different treatment groups after 24 h intravenous injection. Similarly, the blood examination also demonstrated no significant toxic side effects in the BTP treatment group after 24 h post-injection. Correspondingly, the major organs of the mice were collected for H&E staining analysis. Consistent with the above results, no obvious damage was observed in the organs after various treatments (Supplementary Figure S15). These results collectively indicate that BTP exhibits high biosafety without potential acute toxicity after systemic injection. Additionally, the potential neurotoxic effects of HD-MTX and ALB-phage were assessed. As displayed in Fig. 5h, HD-MTX treatment triggered high levels of neuroinflammation and toxicity-related inflammatory cytokines (TNF-α, IL-6 and IL-1β). A limited increase in inflammatory cytokines was found in the L-Pak and ALB-phage treatment groups, suggesting that the targeted delivery of low-dose MTX mediated by BTP could significantly reduce the neurotoxicity caused by high-dose chemotherapy. The same result was also confirmed by H&E staining of brain tissues (Fig. 5i). After treatment with HD-MTX, the intercellular spaces in the hippocampus widened, and vacuolization was observed. In contrast, the hippocampal cells are closely packed, with clearly defined edges in L-Pak and ALB-phage treatment groups, indicating a reduction in neurotoxicity induced by MTX. Overall, these results demonstrate that BTP-mediated low-dose MTX treatment exhibits low toxicity and high therapeutic efficacy for CNS-related diseases.

### Enhanced anti-leukemia effects of ALB-phage in the CNS

**Fig. 6:**
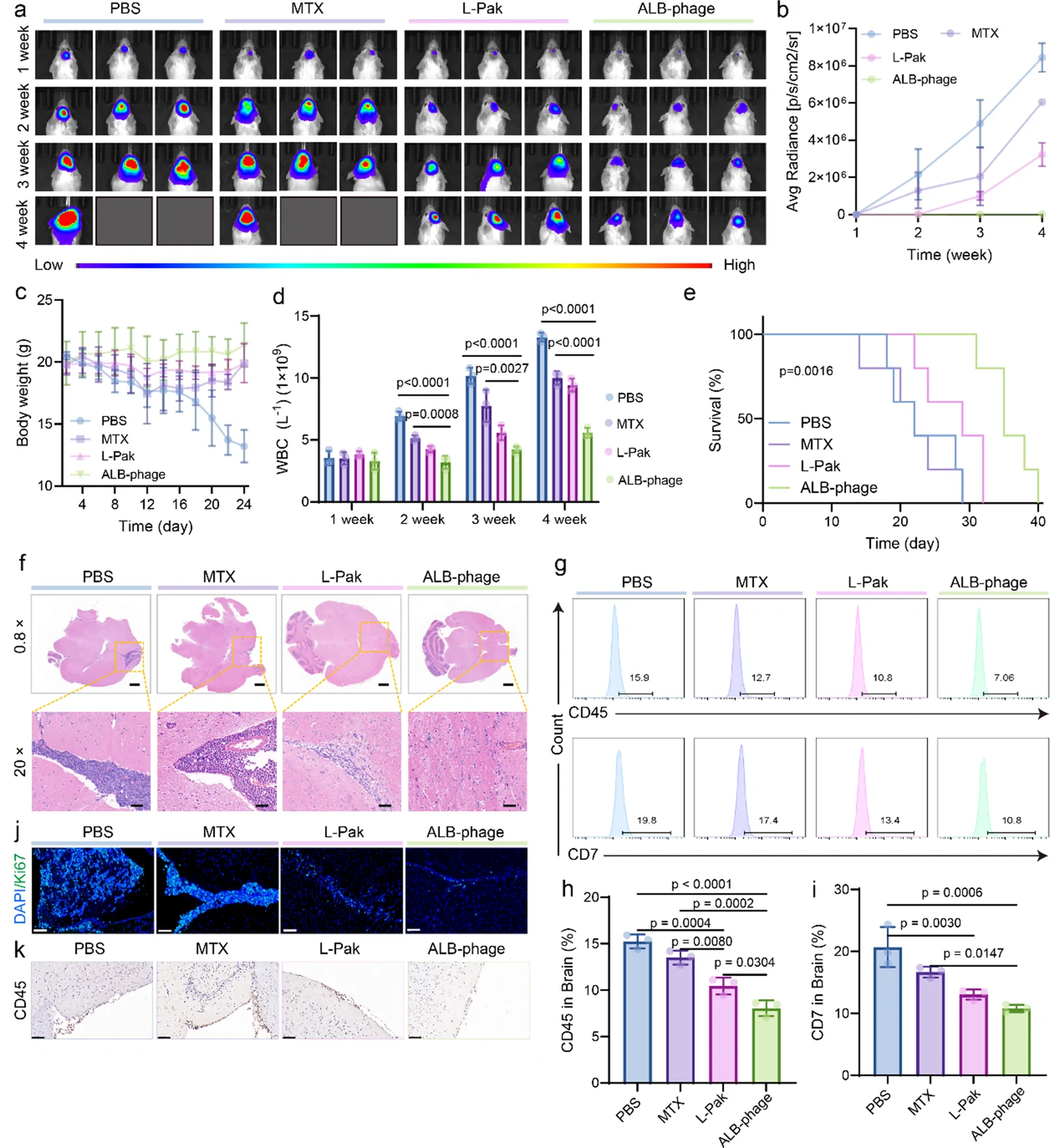
Evaluation of the anti-leukemia effect of ALB-phage. (**a**-**b**) Representative IVIS images (three mice per group are shown) and quantification of bioluminescence intensity of CNS leukemia mice after various treatments for leukemia burden monitoring (*n* = 5). (**c**) Body weight of CNS leukemia mice after receiving different treatments (*n* = 5). (**d**) WBC counts in PB of CNS leukemia mice after various treatments (*n* = 3). (**e**) CNS leukemia mice survival curve during the therapy (*n* = 5). (**f**) H&E staining of the brain tissue of CNS leukemia mice after treatment with PBS, MTX, L-Pak and ALB-phage (Scale bar: 1 mm in 0.8 × and 50 μm in 20 ×). (**g**-**i**) Representative FCM images and quantitative analysis of the CD45 and CD7 leukemia blasts in the brain tissues of CNS leukemia mice (*n* = 3). (**j**-**k**) Immunofluorescence staining of Ki67 and immunohistochemical staining of CD45 of the brain tissues in CNS leukemia mice after different treatments (Scale bar: 100 μm). Data are presented as mean ± SD. Statistical analysis in (**d**, **h**, **i**) was determined by one-way ANOVA with Tukey’s post-hoc test, and the survival curve in (**e**) was analyzed by the log-rank (Mantel–Cox) test

To evaluate the anti-leukemia effects of BTP-guided L-Pak, an orthotopic CNS leukemia mouse model was established by in situ injection of luciferase-transfected MOLT-4 cells (MOLT-4-Luc). Four days after injection, the mice were randomly divided into four groups and administered the following treatments: PBS, MTX, L-Pak and ALB-phage every 3 days for a total of five doses. The progression of the tumor was monitored through weekly IVIS imaging. As shown in Fig. 6a-b, the bioluminescence intensity of brain tumors in the PBS and MTX treatment groups increased rapidly by the second week compared to other groups. By the fourth week, mice in both treatment groups began to die. L-Pak treatment showed significant tumor suppression effects during the first two weeks. However, tumor proliferation accelerated rapidly from the third week. In contrast, ALB-phage exhibited a marked tumor suppression effect. Although tumor bioluminescence intensity increased in the fourth week, it still demonstrated the lowest tumor burden compared to the other treatment groups. Accordingly, due to the higher tumor burden, the body weight of mice in the PBS treatment group decreased significantly compared to the other treatment groups (Fig. 6c). Besides, the changes in white blood cells (WBC) in the peripheral blood were also continuously monitored during the treatment. In line with the above results, the WBC levels were the lowest in the ALB-phage treatment group. Compared with the PBS group, it decreased by 53.9%, 58.4%, and 58% in two, three and four weeks, respectively (Fig. 6d). Specifically, the survival curve of the mice also confirmed that ALB-phage significantly increased survival and prolonged the survival period from 29 days to 40 days with a survival increase of 37.9% (Fig. 6e). Moreover, to further verify the anti-leukemia effects of ALB-phage treatment, the brain tissues and major organs of mice from various treatment groups were harvested for leukemic blast analysis. As shown in Fig. 6f and Supplementary Figure S16, PBS and MTX treatment groups showed significant cellular structure disruption and tumor infiltration. Liver metastasis of tumors was also observed in the PBS treatment group. In contrast, the tumor infiltration level in the L-Pak treatment group was lower, suggesting that nanovesicles improved both leukemic cell killing and the non-specific damage induced by MTX. Notably, ALB-phage exhibited the most effective inhibition of tumor proliferation and minimized brain tissue damage, suggesting that BTP could effectively enhance drug delivery across the BBB while reducing toxicity to normal brain tissue by selectively targeting tumors. The same result was also confirmed by FCM analysis. As shown in Fig. 6g-i, the levels of CD45^+^ WBC in the brain tissue were the highest in the PBS group. It was effectively reduced by 47.2% (to PBS), 40.4% (to MTX), and 22.9% (to L-Pak), respectively, after ALB-phage treatment. The CD7^+^ leukemia cell levels also decreased by 47.8% (to PBS), 35.2% (to MTX), and 17.1% (to L-Pak), respectively. Furthermore, the Ki67 immunofluorescence staining of brain tissue also revealed that ALB-phage treatment effectively inhibited the proliferation of leukemia cells (Fig. 6j). Immunohistochemical staining showed that CD45 leukemia cells infiltrated from the meninges into the brain parenchyma. This invasion process was significantly blocked in the ALB-phage treatment group (Fig. 6k).

## Conclusions

The blood–brain barrier (BBB) represents the primary obstacle to effective treatment of central nervous system (CNS)–related diseases. To address the current limitations of clinically used CNS leukemia therapeutics, there is an urgent need to develop noninvasive, targeted drug-delivery strategies capable of crossing the BBB and delivering low-dose methotrexate (MTX) to enhance anti-leukemia efficacy in the brain. In this study, we developed a bioengineered bacteriophage-based nanovesicle backpack with preferential BBB-penetrating and leukemia cell–targeting capabilities for CNS leukemia therapy. Encapsulation of MTX within this system resulted in enhanced anti-leukemia effects at low MTX doses, as demonstrated in both in vitro and in vivo assays. Mechanistically, BTP-guided nanovesicles exhibited efficient BBB-crossing effect predominantly *via* energy-dependent macropinocytosis, with an additional contribution from clathrin-mediated endocytosis. Notably, the L-Pak backpack showed specific cellular binding in an in vitro BBB model, as well as efficient CNS targeting and accumulation in an orthotopic CNS leukemia mouse model following intravenous administration. Owing to the strong BBB-penetrating capacity of BTP bacteriophages, the L-Pak backpack effectively eliminated leukemia cells using low-dose MTX while maintaining favorable biosafety, thereby avoiding the neurotoxicity associated with high-dose MTX. Consequently, treatment with ALB-phage significantly prolonged both survival rate and survival time in CNS leukemia mice, highlighting this platform as a promising alternative strategy for improving CNS leukemia therapy in clinical settings.

While this study demonstrates compelling therapeutic efficacy in preclinical models, several challenges must be addressed before clinical translation. First, as a bacteriophage-derived carrier, M13 is potentially immunogenic. Repeated intravenous administration may elicit anti-phage neutralizing antibodies. In addition, pre-existing immunity in humans could accelerate clearance and reduce repeat-dose efficacy. Systematic profiling of humoral and cellular responses is therefore needed. Rigorous, long-term evaluations of in vivo biosafety, pharmacokinetics, and immunogenicity will also be essential for both the bacteriophage and the cell membrane–derived components. Second, clinical translation will also depend on reproducible, scalable manufacturing. This requires standardized bacteriophage amplification and purification with strict endotoxin control, as well as uniformity of the liposomal MTX backpack. Moreover, a consistent, GMP-compatible source of leukemia cell membranes is needed to ensure reliable homotypic targeting.

Beyond CNS leukemia, the highly modular and programmable nature of bacteriophage-based nanocarriers positions this platform as a broadly adaptable strategy for treating a wide spectrum of CNS disorders and other diseases requiring efficient traversal of physiological barriers. Looking forward, the integration of living or biohybrid materials into precision drug-delivery systems may enable a new generation of multifunctional theranostic platforms, thereby expanding the horizons of disease diagnosis and treatment and accelerating the clinical translation of barrier-crossing nanomedicines.

## Experimental section

### Materials

Methotrexate (MTX), lecithin, cholesterol, EDC and NHS were purchased from Macklin. 1,2-distearoyl-sn-glycero-3-phosphoethanolamine-N-[succinimidyl(polyethylene glycol)] (DSPE-PEG_2000_-NHS) was bought from Xi’an Ruixi Biological Technology Co., Ltd. 2-deoxy-D-glucose (2-DG), chlorpromazine and wortmannin were purchased from Selleck. FITC anti-human CD7 (Clone CD7-6B7) and APC anti-human CD45 (Clone 2D1) antibodies were purchased from BioLegend. Cell Counting Kit-8 (CCK-8), Annexin V-FITC/PI Apoptosis and Necrosis Detection Kit, Cell Cycle and Apoptosis Analysis Kit, Propidium Iodide (PI) and Calcein AM were provided by Beyotime Biotechnology Co. Ltd. ELISA kits for TNF-α, IL-6 and IL-1β were bought from Ruixin Biotechnology Co., Ltd. (Quanzhou, China). M13 phages were purchased from Wuhan GeneCreate Biological Engineering Co., Ltd.

### Cell lines and animals

Human acute lymphoblastic leukemia cells MOLT-4, Jurkat and CCRF-CEM and mouse brain microvascular endothelial cells (BMVECs) were purchased from Procell Life Science&Technology Co. Ltd. Luciferase-transfected MOLT-4 cell (MOLT-4-Luc) was provided by Genomeditech Co. Ltd. (Shanghai, China). MOLT-4, Jurkat, and CCRF-CEM cells, as well as BMVECs, were cultured in RPMI 1640 and DMEM medium containing 10% fetal bovine serum (FBS; Invitrogen), penicillin (100 U/mL; Invitrogen), and streptomycin (100 μg/mL; Invitrogen) in an incubator at 37 °C with 5% CO_2_, respectively. For the establishment of tumor-bearing mouse models, B-NDG (NOD.CB17-*Prkdc*^*scid*^*Il2rg*^*tm1*^/Bcgen, female, 6–8 weeks old) (Biocytogen Pharmaceuticals Co., Ltd., Beijing) mice were used to construct an orthotopic CNS leukemia mouse model. For in vivo biosafety evaluation, BALB/c mice (female, 6–8 weeks old) were obtained from Charles River Laboratories (Beijing, China).

### Engineering BTP phages

To improve the affinity of WT M13 phages to CNS, a BBB-targeted peptide sequence (CFFWKFRWMC) was inserted into the N-terminus of the pⅢ protein of WT phages. The target sequence was TGTTTCTTCTGGAAATTCCGTTGGATGTGT. M13KE was used as the vector and *SnaBI* and *BamHI* were used for restriction. The engineered M13 phages that stably expressed the target peptide were verified by DNA sequencing.

### Fabrication and characterization of L-Pak

Firstly, MTX@Lipo was prepared by using the thin-film hydration method. L-α phosphatidylcholine (1 mg), cholesterol (0.25 mg), and DSPE-PEG_2000_-NHS (0.02 mg) were dissolved in ethanol. After the thin lipid film was formed using rotary evaporation, a HEPES buffer solution containing MTX was added, and ultrasonication was performed for 30 min to form MTX-encapsulated liposomes (MTX@Lipo). To prepare nanovesicles, leukemia cell membranes were ultrasonically mixed with MTX@Lipo at a mass ratio of 4:1, and then incubated at 37 °C for 1 h to promote membrane fusion. Subsequently, the obtained nanovesicles were successively extruded to obtain L-Pak with uniform sizes.

The particle size and zeta potential of different nanoparticles were measured by a laser particle size analyzer (Nicomp Z3000). The morphologies of nanovesicles and bacteriophages were observed using transmission electron microscopy (TEM) (JEOL JEMF200). The synthesis of DSPE-PEG_2000_-PLGVR was characterized by a Fourier transform infrared spectrometer (FT-IR) (Thermo Scientific Nicolet iS20). The membrane proteins were identified by SDS-PAGE and Western blot analysis.

### Drug release behavior and stability evaluation

To evaluate the release behavior of ALB-phage at CSF in response to matrix metalloproteinase-9 (MMP-9), 2 mg/mL ALB-phage was placed in the dialysis bag (MWCO: 3000), and incubated in CSF and PBS buffer solutions, with or without the addition of MMP-9 at 37 °C. At the indicated time points, appropriate amounts of dialysate were harvested from the buffer solution, and the drug concentration of MTX was detected by high-performance liquid chromatography (HPLC).

For stability of the assembly determination, the L-Pak was placed in the culture medium containing FBS, and the particle size was measured at different times. Furthermore, the surface potential of ALB-phage was also measured at the indicated time points. To more intuitively demonstrate the stability of the system, ALB-phage was placed in PBS and the culture medium, respectively, and then imaged on different days.

### Investigate the in vitro BBB-crossing efficiency

For the evaluation of the BBB targeting effect of BTP, Cy5.5-labeled WTP and BTP were added and incubated with BMVEC cells for 4 h. Then, the BMVEC cells were collected for confocal laser scanning microscope (CLSM) imaging and flow cytometry (FCM) analysis. Meanwhile, the efficiency of transcellular transport was further analyzed by fluorescence intensity quantification and IVIS imaging. Briefly, the 8 μm transwell chamber was first pre-coated with a layer of gelatin as the base layer. Then, BMVEC cells were inoculated onto the gelatin substrate at a density of 1 × 10^5^ cells per well. After 48–72 h of culture, Cy5.5-labeled WTP and BTP were added. At the indicated time, the solutions in the bottom chamber were collected for fluorescence intensity measurement. Also, fluorescence imaging by the IVIS system was performed on the transwell and the well plate respectively.

To evaluate the ability of the nano-backpack to cross the BBB guided by the BTP bacteriophages, an in vitro BBB co-culture model was established. In brief, the 8 μm transwell chamber was first pre-coated with a layer of gelatin as the base layer. Then, BMVEC cells were inoculated onto the gelatin substrate at a density of 1 × 10^5^ cells per well. MOLT-4 cells were inoculated in the bottom chamber of the 6-well plate. BMVEC cells were allowed to grow for 48–72 h to achieve fusion. After that, FITC-labeled L-Pak and Cy5.5-labeled BTP were added to the upper chamber for BBB crossing. After 6 h of incubation, BMVEC and leukemia cells were collected separately for CLSM imaging and FCM analysis.

### Exploration of the transcellular transport mechanism mediated by BTP

BMVEC cells were seeded into a 6-well plate at a density of 1 × 10^6^/ well. After 24 h of culture, the cells were treated with Cy5.5-labeled BTP and then cultured at 4 °C and 37 °C for an additional 2 h. Then, the BMVEC cells were collected and washed three times with PBS. The intracellular fluorescence intensity was analyzed using CLSM and FCM analysis. Moreover, a panel of endocytosis inhibitors, including 2-deoxy-D-glucose (2-DG) (1 mM), chlorpromazine (30 µM), and wortmannin (10 µM), was added and pre-incubated with BMVEC cells for 1 h. Then, the Cy5.5-labeled BTP was added and cultured for another 6 h. The intracellular fluorescence intensity was analyzed using CLSM and FCM analysis.

### In vitro cell cytotoxicity, cell cycle arrest and apoptosis evaluation

For cell cytotoxicity assays, MOLT-4, Jurkat, and CCRF-CEM cells were seeded into a 96-well plate at a density of 5 × 10^4^/ well. Then, cells were treated with PBS, BTP, MTX, MTX@Lipo, L-Pak, and ALB-phage, individually. Furthermore, to study the responsiveness of MMP-9, the MOLT-4 cells were treated with ALB-phage without or with an MMP-9 inhibitor. After 24 h of incubation, CCK-8 was added to the above cells and incubated at 37 °C for 3 h. The cell viability was determined by measuring the absorbance at 450 nm. Besides, the cytotoxicity was further demonstrated by PI/Calcein AM staining to show the ratio of live/dead cells.

For cell cycle and apoptosis determination, MOLT-4 cells were inoculated into 6-well plates at a density of 1 × 10^6^/ well for 24 h. Then, cells were treated with PBS, BTP, MTX, L-Pak and ALB-phage. After 24 h of incubation, the cells were collected for analysis. Cell cycle arrest was analyzed by PI staining according to the cell cycle and apoptosis analysis kit. Cell apoptosis was determined by Annexin V-FITC/PI staining and immunofluorescence staining of caspase-3. For cell proliferation evaluation, immunofluorescence staining of PCNA was performed.

### In vivo brain targeting and biosafety effects

B-NDG mice were intracranially injected with 1 × 10^4^ MOLT-4-Luc cells to establish the CNS leukemia model. For the evaluation of the brain-targeting effect of bacteriophages, CNS leukemia mice were randomly divided into three groups. Then, Cy5.5-labeled WTP and BTP were intravenously injected into mice. Free Cy5.5 was used as a control group for small-molecule drugs. The biodistribution of Cy5.5-labeled bacteriophages was monitored by the IVIS imaging system at the indicated time points. Similarly, the biodistribution of Cy5.5-marked L-Pak and ALB-phage was evaluated by the IVIS imaging system. 6 h after the injection, the major organs (heart, liver, spleen, lung, and kidney) and brains of the mice were collected for ex vivo fluorescence imaging. Additionally, the Cy5.5 fluorescence in the brain was also further analyzed through immunofluorescence staining.

For pharmacokinetic analysis of MTX, CNS leukemia mice were randomly divided into three groups and treated with MTX, L-Pak and ALB-phage, respectively. Peripheral blood (PB) samples from mice were collected at the indicated time points (0 h, 0.25 h, 0.5 h, 1 h, 2 h, 4 h, 6 h, 8 h, 12 h and 24 h). The plasma MTX concentration was determined by HPLC.

For in vivo biosafety measurement, healthy BALB/c mice were treated with PBS, BTP, L-Pak and ALB-phage, respectively. After 24 h of intravenous injection, PB samples were collected from the mice to examine changes in blood cells, as well as liver and kidney functions. To further investigate the toxicity effect of HD-MTX, the serum cytokines TNF-α, IL-6 and IL-1β were detected by enzyme-linked immunosorbent assay (ELISA). The neurotoxicity was evaluated by H&E staining of brain tissues after various treatments.

### In vivo anti-leukemia effect

4 days after MOLT-4-Luc cells were inoculated orthotopically, CNS leukemia mice were randomly divided into 4 groups and received the following treatments, including PBS, MTX, L-Pak and ALB-phage every three days for a total of five doses. Tumor progression was monitored through weekly bioluminescence imaging by using an IVIS imaging system. The weight and survival time of the mice during the treatment were recorded every other day. Moreover, the counts of white blood cells (WBC) in the PB were examined by blood routine measurement every week to evaluate the leukemia blasts. After four weeks of treatments, three mice were randomly sacrificed in each treatment group, and the major organs and brains of the mice were harvested for H&E staining, immunofluorescence staining of Ki67, and immunohistochemical staining of CD45 for evaluating leukemia infiltration. In addition, the CD45 and CD7-positive leukemia cells in the brain were further analyzed through FCM.

### Statistics analysis

All quantitative data were presented as means ± SD. For normally distributed data sets with equal variances, statistical analysis for comparing two experimental groups was performed using an unpaired t-test, and one-way ANOVA with Tukey’s post hoc analysis was carried out for multi-group comparison. Survival curves were analyzed with the log-rank Mantel-Cox test. Statistical analysis was carried out using GraphPad Prism 10. FlowJo was used to analyse the data of flow cytometry.

## Supplementary Information

Below is the link to the electronic supplementary material.

Supplementary Material 1

## Data Availability

No datasets were generated or analysed during the current study.

## Abbreviations

ALB-phage: Anti-leukemia backpack–phage
ALL: Acute lymphoblastic leukemia
BBB: Blood–brain barrier
BMVEC: Brain microvascular endothelial cell
BTP: Brain-targeting phage
CCK-8: Cell Counting Kit-8
CDX: Cell-line-derived xenograft
CLSM: Confocal laser scanning microscopy
CNS: Central nervous system
CSF: Cerebrospinal fluid
DHFR: Dihydrofolate reductase
DLS: Dynamic light scattering
DMEM: Dulbecco’s modified Eagle medium
DSPE-PEG₂₀₀₀: 1,2-distearoyl-sn-glycero-3-phosphoethanolamine-N-[(polyethylene glycol)-2000
EDC: 1-ethyl-3-(3-dimethylaminopropyl)carbodiimide
ELISA: Enzyme-linked immunosorbent assay
FBS: Fetal bovine serum
FCM: Flow cytometry
FITC: Fluorescein isothiocyanate
FRET: Förster resonance energy transfer
FT-IR: Fourier-transform infrared spectroscopy
H&E: Hematoxylin and eosin
HD-MTX: High-dose methotrexate
HPLC: High-performance liquid chromatography
IL-1β: Interleukin-1β
IL-6: Interleukin-6
IVIS: In vivo imaging system
LCM: Leukemia cell membrane
L-Pak: Leukemia-targeting backpack
MMP: Matrix metalloproteinase
MMP-9: Matrix metalloproteinase-9
MTX: Methotrexate
NGS: Next-generation sequencing
NHS: N-hydroxysuccinimide
PB: Peripheral blood
PBS: Phosphate-buffered saline
PDI: Polydispersity index
PI: Propidium iodide
RPMI 1640: Roswell Park Memorial Institute 1640 medium
HEPES: 4-(2-hydroxyethyl)-1-piperazineethanesulfonic acid
MWCO: Molecular weight cut-off
SDS-PAGE: Sodium dodecyl sulfate–polyacrylamide gel electrophoresis
TEM: Transmission electron microscopy
TNF-α: Tumor necrosis factor-α
UV-vis: Ultraviolet–visible spectroscopy
WBC: White blood cell
WTP: Wild-type phage
