# Supplementary material for: Biohybrid M13 phage backpacks for active blood–brain barrier traversal and precision central nervous system leukemia treatment
Source: Biomark Res. 2026 Sep 16;14:123. doi: 10.1186/s40364-026-01000-3 (PMC13579763; doi:10.1186/s40364-026-01000-3)
Supplement: Supplementary file 1 [file 40364_2026_1000_MOESM1_ESM.docx]

Supplementary Information

**Biohybrid M13 Phage Backpacks for Active Blood–Brain Barrier Traversal and Precision Central Nervous System Leukemia Treatment**

Wei Wu^1,4^, Cheng-Ling Zhang^1,2,3^, Yu-Ling Yang^1,2,3^, Xiaoyuan Chen^5*^, Xi Zhang^1,2,3*^ and Xue Dong^1,2,3*^

^1^Medical Center of Hematology, Xinqiao Hospital of Army Medical University, Chongqing 400037, P. R. China.

^2^Chongqing Key Laboratory of Hematology and Microenvironment. Chongqing 400037, P. R. China.

^3^State Key Laboratory of Trauma and Chemical Poisoning, Chongqing 400037, P. R. China.

^4^School of Life Science, Chongqing University, Chongqing, 400044, P. R. China.

^5^Shandong Provincial Key Laboratory of Precision Oncology, Shandong Cancer Hospital and Institute, Shandong First Medical University and Shandong Academy of Medical Sciences, Jinan, 250117, China.

*Corresponding author, E-mail: chen9647@gmail.com (X.C.); zhangxxi@sina.com, zhangxi@tmmu.edu.cn (X.Z.); dongx1203@163.com, xuedong13@tmmu.edu.cn (X.D.).

**Supplementary Figure S1.** The plasmid map of engineered BTP bacteriophages.

**Supplementary Figure S2.** NGS sequencing of five clones of the p3 protein of the engineered BTP bacteriophages.

**Supplementary Figure S3.** Quantitative fluorescence analysis of major organs (n = 3).

**Supplementary Figure S4.** Representative FCM images and quantitative analysis after BMVEC cells treatment with different incubation temperatures and endocytosis inhibitors (n = 3).

**Supplementary Figure S5.** Preparation and characterization of DSPE-PEG_2000_-PLGVR conjugations. (a) The synthesis scheme of DSPE-PEG_2000_-PLGVR. (b) FT-IR spectrum of DSPE-PEG_2000_-PLGVR. (c) H-NMR spectrum of PLGVR peptide and DSPE-PEG_2000_-PLGVR.

**Supplementary Figure S6.** Hydrolyzed particle size and zeta potential of nanovesicles formed by the fusion of LCM at different ratios with liposomes. (a) 4:1, (b) 5:1, (c) 10:1, (d) 20:1.

**Supplementary Figure S7.** UV-vis absorption spectrum of Liposome, BTP, MTX, L-Pak and ALB-phage.

**Supplementary Figure S8.** Stability images of L-Pak and ALB-phage in PBS and culture medium.

**Supplementary Figure S9.** Cell viability of BMVEC cells after treatment with different concentrations of BTP bacteriophages (n = 6).

**Supplementary Figure S10.** Cell viability of MOLT-4 cells after treatment with different concentrations of MTX, L-Pak and ALB-phage (n = 6).

**Supplementary Figure S11.** Calcein AM/PI staining of MOLT-4 cells after different treatments.

**Supplementary Figure S12.** Typical cell cycle FCM images of MOLT-4 cells after various treatments (n = 3).

**Supplementary Figure S13.** Western blotting analysis of the protein expression of MOLT-4 cells after various treatments.

**Supplementary Figure S14.** IVIS fluorescence images and quantitative analysis of major organs after Cy5.5, L-Pak and ALB-phage treatments in CNS leukemia mice (n = 3).

**Supplementary Figure S15.** H&E staining of major organs in balb/c mice after various treatments (Scale bar: 100 μm).

**Supplementary Figure S16.** H&E staining of major organs in CNS leukemia mice after various treatments (Scale bar: 100 μm).
